# Psychosocial skills of ICU healthcare staff providing care to patients with COVID-19

**DOI:** 10.1192/j.eurpsy.2022.1932

**Published:** 2022-09-01

**Authors:** M. Soussi, M. Kahloul, I. Kacem, M. Ajmi, Y. Slama, A. Harzali, A. Chouchane, M. Maoua, N. Mrizak, W. Naija

**Affiliations:** 1 Sahloul Academic Hospital, University of medicine, “Ibn Al Jazzar”, Sousse, Tunisia, Department Of Anesthesia And Intensive Care,, Sousse, Tunisia; 2 Farhat Hached Academic Hospital, Occupational Medicine, Sousse, Tunisia

**Keywords:** Covid-19, Psychosocial skills, healthcare staff

## Abstract

**Introduction:**

The COVID-19 crisis has imposed deep improvements in ICU responsiveness face to unprecedented and uncertain situations. In addition to strengthening logistics resources, this responsiveness required the development of psychosocial skills of healthcare providers, especially in ICU.

**Objectives:**

To assess extrinsic factors interfering with psychosocial skills of the staff working in COVID-19 ICU and to analyze the different dimensions of these skills.

**Methods:**

This is an observational descriptive study conducted at the COVID-19 ICU of an Academic Hospital, during a one-month period. All healthcare providers were enrolled. Data collection was based on a self-administered questionnaire including: socio-demographic factors; the general perception of work in covid-19 ICU and psychosocial skills. Six dimensions were explored separately, then by a standardized scale ranging from 0 to 100.Three levels of satisfaction were considered.

**Results:**

Fifty-five healthcare providers were enrolled. The average age was 32 years. The sex ratio was 0.25. Mean scales of satisfaction were 53.6 for professional status and occupational security; 62.4 for working conditions and 69.8 for relational aspects. The most altered extrinsic factors were satisfaction regarding the salary and satisfaction regarding the administration policy with mean scores of 15 and 10 respectively. Satisfying psychosocial skills were creative and critical thoughts, self-awareness and empathy for others, communication and interpersonal relationships. Whereas the most impaired skills were stress management and problems solving, with mean scales of 49.6 and 68.3 respectively.

**Conclusions:**

Psychosocial skills were generally acceptable. However, they could be improved by specific actions targeting extrinsic factors.

**Disclosure:**

No significant relationships.

